# The Australian dingo: untamed or feral?

**DOI:** 10.1186/s12983-019-0300-6

**Published:** 2019-02-13

**Authors:** J. William O. Ballard, Laura A. B. Wilson

**Affiliations:** 1 School of Biotechnology and Biomolecular Science, University of New South Wales, Sydney, NSW 2052 Australia; 2 School of Biological, Earth and Environmental Sciences, University of New South Wales, Sydney, NSW 2052 Australia

**Keywords:** Unconscious selection, Artificial selection, Domestication, Canid, Hybridization

## Abstract

**Background:**

The Australian dingo continues to cause debate amongst Aboriginal people, pastoralists, scientists and the government in Australia. A lingering controversy is whether the dingo has been tamed and has now reverted to its ancestral wild state or whether its ancestors were domesticated and it now resides on the continent as a feral dog. The goal of this article is to place the discussion onto a theoretical framework, highlight what is currently known about dingo origins and taxonomy and then make a series of experimentally testable organismal, cellular and biochemical predictions that we propose can focus future research.

**Discussion:**

We consider a canid that has been unconsciously selected as a *tamed* animal and the endpoint of methodical or what we now call artificial selection as a *domesticated* animal*.* We consider wild animals that were formerly tamed as *untamed* and those wild animals that were formerly domesticated as *feralized*. Untamed canids are predicted to be marked by a signature of unconscious selection whereas feral animals are hypothesized to be marked by signatures of both unconscious and artificial selection. First, we review the movement of dingo ancestors into Australia. We then discuss how differences between taming and domestication may influence the organismal traits of skull morphometrics, brain and size, seasonal breeding, and sociability. Finally, we consider cellular and molecular level traits including hypotheses concerning the phylogenetic position of dingoes, metabolic genes that appear to be under positive selection and the potential for micronutrient compensation by the gut microbiome.

**Conclusions:**

Western Australian Government policy is currently being revised to allow the widespread killing of the Australian dingo. These policies are based on an incomplete understanding of the evolutionary history of the canid and assume the dingo is feralized. However, accumulated evidence does not definitively show that the dingo was ever domesticated and additional focused research is required. We suggest that incorporating ancient DNA data into the debate concerning dingo origins will be pivotal to understanding the evolutionary history of the canid. Further, we advocate that future morphological, behavioural and genetic studies should focus on including genetically pure Alpine and Desert dingoes and not dingo-dog hybrids. Finally, we propose that future studies critically examine genes under selection in the dingo and employ the genome from a wild canid for comparison.

**Electronic supplementary material:**

The online version of this article (10.1186/s12983-019-0300-6) contains supplementary material, which is available to authorized users.

## Background

Canids are among the most widely distributed carnivores, with at least one species present on every continent except Antarctica. Undisputedly, the dingo is Australia’s wild dog and top-order predator. Colloquially, it is considered a “*lightning- rod*” of the land as it generates polarised opinions from Aboriginal people, pastoralists, tourism operators, conservationists, ecologists and evolutionary biologists. Here, we do not attempt to reconcile all the disparate views. Rather, we aim to place the discussion of dingo origins onto a theoretical framework, highlight what is currently known and what is posited about dingo origins and taxonomy. We then make a series of experimentally testable organismal, cellular and biochemical predictions that we hope will focus future research and determine whether dingo ancestors were ever domesticated.

The dingo is a common feature in Australian Aboriginal peoples dreamtime stories, which are an important part of the indigenous culture, spiritualism and oral history [1]. One example is a Cape York dreamtime story of the Giant Devil Dingo who becomes Aboriginal peoples friend and helper [2]. In western New South Wales, the painted tracks of a human and kangaroo (without any associated dingo tracks) tell of the folly of a hunter who fails to take his dingo with him and consequently loses his prey [3]. However, dingoes are also known to attack sheep and are therefore not well-respected by many Australian pastoralists. Widespread reforms to the Western Australian Biodiversity Conservation act are expected in 2019. In a statement to the Australian Broadcasting Corporation, Western Australia’s Minister for the Environment said he “*will make an order that determines that the dingo is not fauna for the purposes of the act*”. This will mean that dingoes can be trapped or killed without permission in many places. As the state of Western Australia covers the western third of the continent this legislation has the potential to decimate the dingo population.

Ecotourists travel from around the globe to view the dingo on Fraser Island and in dingo sanctuaries such as the Bargo Dingo Sanctuary, in New South Wales. Fraser Island is the largest sand island in the world and caters to more than 300,000 visitors annually. The dingo population is estimated to be between 150 and 200 animals and their conservation is of national significance [4]. Concerns have long been expressed about the potential for dangerous interactions between dingoes and humans. On April 30, 2001, dingoes mauled a 9-year-old boy to death and the public demanded firm management actions. However, the fundamental question remained. Do we manage the people or the animals? Public opinion was polarised. More recently in 2017, two dingoes on Fraser Island were destroyed due to high risk interactions with visitors, while three died in vehicle strikes [5]. To date in 2018, there have been more than 17 reports of interactions between dingoes and people on Fraser Island [5].

The present-day ecological role of the dingo is debated [6]. It is intimately involved in the ecological functioning of healthy native habitats suggesting it has been present on the continent for a lengthy period [7–9]. Further, as top predator the dingo plays an important role in regulating herbivore populations, such as kangaroos [10–13]. There is considerable debate, however, whether the dingo influences the numbers of introduced red foxes or caused the extinction of the Tasmanian tiger on mainland Australia [14–17].

One issue we do not debate is the binomial nomenclature of the dingo. We acknowledge that there are differences of opinion on this matter, but suggest that it is only when consensus is reached as to whether the dingo was ever domesticated that the debate on dingo taxonomy can logically proceed. In this article we simply refer to the canid as the Australian dingo. Currently, the alternatives being debated include *Canis dingo*, *Canis familiaris*, *Canis lupus dingo* and *Canis familiaris dingo* [18–21].

## Discussion

Here, we first consider the process of domestication as a framework to distinguish between alternative hypotheses. The degree to which tamed-like and domestic-like traits are found in free-living canines depends on the trajectory and strength of selection at the point along the domestication continuum where the animal became free-living. We then review the movement of dingo ancestors into Australia and suggest that it has likely interacted with humans for over 5000 years. We consider dingo whole organism level traits of skull morphometrics, brain size, seasonal breeding, and sociability and make predictions that will facilitate determination of whether the dingo was ever truly domesticated. In the final section, we discuss cellular and molecular level traits including the disparate views on phylogenetic position of the dingo relative to primitive domestic dogs such as the African Basenji. One clear prediction is that dingoes are expected to show a genetic signature of an amylase duplication if it was historically domesticated, unless there were multiple independent amylase expansions. We conclude that there are at least two dingo ecotypes that we refer to as the Desert and Alpine types, that are likely closely related to New Guinea singing dogs, but the evolutionary position of the Australian dingo relative to domestic dog breeds has not been definitively determined at this time.

### Taming and domestication

**Fig. 1:**
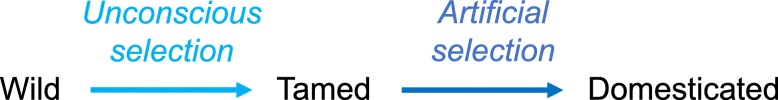
Process of domestication. We define the endpoint of Darwin’s *unconscious* selection as a tamed animal and the endpoint of *methodical,* or what we now call *artificial,* selection as a tamed and domesticated animal*.* Unconscious selection proceeds to make an animal human-friendly without any thought to any predetermined purpose. Artificial selection is the process by which humans selectively develop specific phenotypic traits

While it is not clear why certain species were able to be tamed and domesticated and others not [22], Charles Darwin [23] provides a theoretical framework to begin the discussion (Box 1). Here, we define the endpoint of Darwin’s *unconscious* selection as a tamed animal and the endpoint of *methodical,* or what we now call *artificial,* selection as a tamed and domesticated animal*.* A tamed animal is a wild animal that has been habituated to, and is cared for, in part by humans. Tamed animals may have a causal relationship with humans for example, avoiding humans while breeding but returning for diet supplementation. It is distinct from the relationship of a domesticated animal where humans have a substantial influence over the reproduction of another organism (Fig. 1). Jared Diamond [22] elegantly summarised the difference between tamed and domesticated animals **“***Hannibal’s African war elephants were, and modern Asian work elephants still are, just tamed wild individuals, not individuals of a genetically distinct population born and reared in captivity*”.

In canids, domestication proceeds with the “commensal pathway” mode of domestication [24–26]. This pathway does not typically begin with intentional action to bring animals into the living place of people, rather wild animals are most plausibly attracted to the human niche (food, waste/prey) and enter it of their own accord. Therefore, the initial process likely takes place in the absence of human instigation, and later human-directed selection builds upon the animal already being acquainted with, and able to take advantage of, the human environment.

Wolves are the likely ancestor of dingoes and domestic dogs. Wolf taming likely involved a founder group of less-fearful canids that would have drifted toward nomadic encampments, perhaps to scavenge kills, salvage wounded escapees from the hunt or perhaps people taking pups [27, 28]. Thereafter, these less-fearful wolves may have found utility perhaps as barking sentinels, warning of human and animal invaders approaching at night [27]. Gradually, selection and genetic drift resulting from human activities began to differentiate these wolves from the larger autonomous population. Once people had direct interaction with wolves, a subsequent cultural process involving unconscious selection would have begun. Suitable wolf pups taken as pets would have been socialized to humans and selected for decreased flight behaviour and increased sociality [29], two classical trademarks of tameness (Fig. 1). In parallel it is possible, that some individuals took in wolf pups and this action contributed to the taming of selected canines. Such human induced taming events have been reported to occur in dingoes [30, 31].

Continued artificial selection of tamed canids resulted in domestication [28] (Fig. 1). Nevertheless, there is a surprising lack of agreement on how to define domestication [32], reflecting variation among scholars in their identification of the dichotomy between nature and culture [33]. Beyond acknowledging that it involves a relationship between a domesticate and a domesticator there is little consensus. One general definition of animal domestication describes a gradual process that begins when humans capture and tame an animal that has specific, desired behavioural or physical traits. This definition emphasizes the role of humans in separating the target domesticate from free-living populations [32]. Most generally, it assumes human mastery over reproduction [34], but this may be inadequate for dogs because it implies that people perhaps living 20-40 k years ago [35] intentionally manipulated the reproductive output of wolves. From a developmental perspective, the selection for tameness has been proposed to result in mild developmental deficits in neural crest derived tissues during early development, and these changes have been proposed to underlie the suite of traits associated with domestication [36]. Domestication has also been viewed as a mutualistic process that benefits both domesticate and domesticator [32, 37]. Certainly, this is the case for domestic dogs as they are now likely the most common member of the Carnivora on the planet, which supports the tenet that their relationship with humans has been successful from an evolutionary perspective.

Artificial selection proceeds by removal of the animal from its natural ecological and genetic environments to one where the animal’s maintenance and breeding is controlled by humans [38]. Humans may then select desirable traits from among the domesticated animals and protect them from natural selection. In the narrowest sense, a domesticated animal is one that has been bred in captivity for the purposes of economic profit to a human community that maintains complete mastery over its breeding, organization of territory, and food supply [38]. The advantage for the domesticate is that inter- and interspecific conflicts are reduced and a nutritional source provided.

#### Was the dingo ever domesticated?

**Fig. 2:**
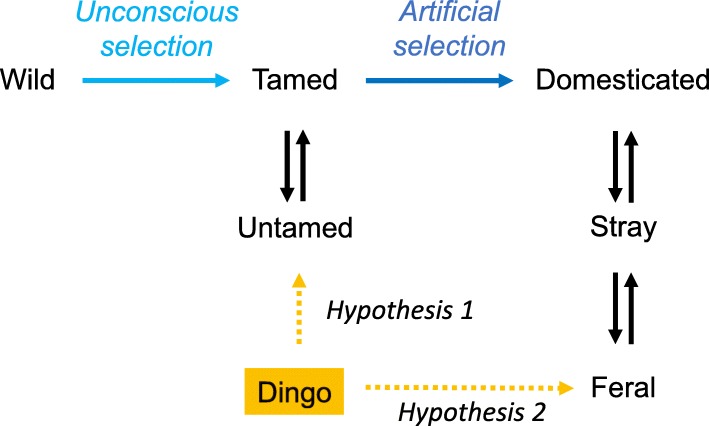
Possible evolutionary position of the dingo. Hypothesis 1 is that the dingo is an untamed dog. Hypothesis 2 is that the dingo is a feralized dog. Untamed animals are predicted to be marked by a signature of unconscious selection whereas feral animals are hypothesized to be marked by a signature of both unconscious and artificial selection

Plausibly, the Australian dingo was tamed to some degree in SE Asia before the arrival of Europeans. We refer to this as *Hypothesis 1*. It seems unlikely that dingoes were domesticated by Australian Aborigines (see discussion below). Gollan [39] critically reviewed the evidence and wrote that dingoes were “*intractible, and unreceptive to the casual attempts by Aborigines to domesticate it*.” Removal of human selection on tamed animals may result in animals returning to the wild (Fig. 2). We know of no specific term that has been used to define a tamed animal returning to the wild, to avoid unnecessary confusion we will simply refer to this event as untaming. If this is true, the dingo has the potential to give unique insights into the processes of domestication [23]. The alternative hypothesis, is that dingoes were tamed and domesticated in SE Asia such that they are now a feral wild canid (Fig. 2). We term this *Hypothesis 2*. We follow Clutton-Brock [40] and define feralized animals as a **“***domesticated animals that return to living in the wild*”. Each stage of the general process of domestication is accompanied by human influence on the environment that changes the trajectory and strength of unconscious and artificial selection. Scientifically, both possibilities are interesting. Politically, there is a titanic divide between these scenarios because some see no difference between individuals that been wild for one generation and a population that has been wild for a thousand (or more) generations.

We posit that when an animal exits the influence of humans and returns to the wild, selected traits that escape selection and drift should leave a mark of the evolutionary history of the animal. Thus, untamed animals would be expected to show organismal and cellular signatures of taming but not domestication while feral animals would be expected to show signatures of both taming and domestication. In this debate we first review the proposed ancestors of the dingo, whose range likely overlapped with wolves. We then consider the types of signatures that may be expected from taming as compared to domestication.

### Evolutionary history of dingoes

Hypothesis 1 (Fig. 2) predicts the wild Asian Grey Wolf is the ancestor of the tamed but undomesticated Pariah dog, which is the ancestor of the dingo. The external morphology of the Pariah dog resembles that of a dingo (Box 2). Phylogenetic analyses of whole genome sequences estimate that dogs and wolves diverged genetically between 36,900 and 41,500 years ago [35, 41]. Further subdivision of dogs into Eastern (Asian) and Western (European and Middle Eastern) groups occurred between 17,000 and 24,000 years ago [35]. Overall, archaeological evidence is scant but the type of dog found in five different archaeological sites of north and central Thailand corresponds to the typical pariah dingo type [42, 43], which is reported to have a “*more informal*” association with people [43], suggestive of taming but not domestication.

Hypothesis 2 (Fig. 2) predicts the Wolf is the ancestor of tamed and domesticated Village Dogs, which are the ancestor of the dingo (Box 3). Village-type dogs are reported have a closer association with people and have been linked with the spread Neolithic farming [43, 44]. Fillios and Taçon [45] and Cairns and Wilton [46], have argued that it is unlikely that dingoes were brought to Australia as part of a Neolithic cultural expansion, as there were no other Neolithic cultural markers (pig, chickens, agriculture) brought to Australia. Nevertheless, demonstration that a village dog was the direct ancestor of the dingo would provide compelling evidence to suggest that the dingo ancestor was domesticated. Therefore, obtaining archaeological data from southeast Asia will be key in understanding the evolutionary history of the dingo. In northern Vietnam there is evidence for domestic dog dated to 4000 cal. BP associated with the Phung Nguyen Culture [47]. One of the most complete village dog specimens comes from Timor -Leste (2967 ± 58 BP) and appears to have been domesticated [48]. Unfortunately, no useful DNA was obtained from this latter specimen at the time, but perhaps the specimen could be revisited with more recent DNA extraction techniques. Unfortunately, hybridization between pariah, village and domestic dogs over the past 5000 years makes it difficult to distinguish these types in extant populations [49].

There are at least two dingo forms, we call ecotypes, that may have colonised Australia independently or may have diverged upon arrival in Australia. These ecotypes are most commonly called Desert and Alpine types. Currently, there is ongoing debate about the uniqueness of the Fraser Island population and a lack of consensus on whether tropical ecotypes exist [46, 50–53]. Dating the divergence times of the Alpine and Desert ecotypes, using complete mitochondrial genomes, suggests the ancestor of the dingo was the undomesticated Pariah dog and not the domesticated Village dog [46, 54]. Cairns and Wilton [46] estimated that the divergence time of the two mtDNA lineages to be 8300 years BP (5742–11,663 95% HPD), which is older than the earliest Neolithic levels in island south east Asia, which date to *c*. 4400 cal. BP [54]. A logical problem with this divergence estimate, however, was that the two dingo lineages were not reported to be monophyletic relative to the New Guinea singing dog. As such, the divergence time may have been incorrectly estimated.

#### Dingoes in Australia

Likely mariners brought canines that became dingoes to Australia [19, 45], possibly as a hunting companion and camp dog or a food source [45]. This method of colonisation resulted in a population bottleneck that reduced genetic variation and makes determination of their history more difficult [55–57]. Clearly, the method of dingo colonization does not even indirectly address whether the canid was tamed or domesticated as a tamed tiger or lion can be transported in a crate. Fillios and Taçon [45] speculated that the Toalean people of Sulawesi and Borneo brought canids to Australia. There are, however, multiple alternate hypotheses including one that suggests dingoes arrived by boat from India [58] and another that they came directly from Taiwan [59]. Again, archaeological samples from SE Asia may help resolve this conundrum.

Dingoes arrived in Australia between 3500 and 12,000 BP. There is no evidence that dingoes have ever inhabited Tasmania, which was separated from Australia by sea level changes approximately 12,000 years ago, strongly suggesting that dingoes did not arrive before this time. Molecular data predicts the dingo lineages diverged 8300 years BP (5742–11,663 95% HPD) [46], however, the oldest confirmed dates of dingoes in southern Australia are between 3348 and 3081 years ago at Madura Cave in Western Australia [14]. Other fossilized dingo remains have been linked to about 3200 years BP at Wombah on the north coast of New South Wales [60], 3000 years BP at Fromm’s Landing in South Australia [61] and 2200 years BP at Thylacine Hole, Western Australia [62].

The dingo exists in Australia as a wild canid, but they may be “*voluntary captive, unmanaged, and with limited functions within the economy or social life of Aborigines*” [39]. Archaeological evidence from dingo specimens excavated from eastern Australia show burials of dingoes in middens, with some of the specimens showing evidence that may imply the existence of breeding populations removed from the wild [63, 64]. Certainly, there is evidence for the taking of pups for pets by Aborigines in many regions of Australia (reviewed by, [31]). The issue, as identified by the Gollan [63], is “*to associate the observed modifications with a trajectory of change towards a domesticated branch of canids*” or conclude that any attempts at breeding of the dingo was more than a “*biologically episodic process*”. Gunn et al. [31] report on the burial of a dingo from Arnhem land plateau and discuss dingo burials and the role of dingoes in Aboriginal beliefs throughout Australia. They conclude that the dingo is typically a companion figure and one that held an extraordinary place in the Aboriginal world and was not “kept” within the confines of the human society. Currently, changes that represent stages in a morphological progression have yet to be identified in extant dingoes. Thus, we conclude that any attempts at breeding dingoes by Aborigines failed to leave descendants and thereby did not influence the evolutionary history of the canid in Australia.

Physical descriptions of the dingo are presented by Smith [65], Crowther et al. [19] and Jackson and colleagues [18]. Briefly, the dingo is described as a medium-sized canine that averages 55 cm tall at the shoulder and 123 cm long. The medium-sized tail is flattish and heavily bushed. The average body mass of a dingo is 15 kg, males being slightly larger than females [66, 67]. The pelage of the dingo is described as short with a hard/dry outer coat and an under coat [65]. Dingoes may have one of five basic coat colours: yellow, brown, ginger/red, black and tan and white [68] with white points (feet, chest and tail tip), however white points are not recorded in early accounts nor are they present in all pre-1900 illustrations or vouchers. Dingoes have erect, pointed ears like wolves. The dingo head is like that of a small wolf, having a narrow muzzle with large canine and strongly developed carnassial teeth and large auditory bullae. As for most wild canines, the presence of a vestigial first digit (‘dew claw’) is infrequent [69]. Clutton-Brock and colleagues [70] observed a single dew claw in one of 15 skins in the British Museum of Natural History.

Corbett [50] mentioned the possibility of three different subspecies of dingo existing in north, central and south-eastern Australia. He tentatively named them as *Canis lupus dingo* Meyer for the Alpine dingo, *Canis lupus macdonnellensis* Matschie for the Desert dingo, and *Canis lupus cobourgensis* Corbett for the Tropical dingo [71]. However, he advised caution on the issue, outlining that subspecific differences could be based on gradients of both rainfall and temperature across the continent, and that therefore populations seemed to overlap frequently. Corbett [51] noted that the dingo skulls from south-eastern Australia were different from those of the rest of the country, but he attributed the differences to hybridization with domestic dogs. Jones [52] agreed that these south-eastern dingoes were morphologically distinct and questioned the validity of applying Corbett’s morphological equations, based on desert populations, to alpine populations. Morphological analysis of fossil dingoes [39] and genetic evidence support the hypothesis that there are two distinct dingoes evolutionary lineages [53, 68, 72], therefore caution needs to be exercised in pooling measurements or studies between the different types.

In this section we have reviewed the movement of dingo ancestors through Asia and into Australia and posit that it has interacted with humans for more than 5000 years. Currently, it is not clear whether the ancestor of the dingo was ever tamed or domesticated, but the weight of evidence currently supports our Hypothesis 1 (Fig. 2). We suggest that obtaining archaeological and DNA data from ancient canids in southeast Asia will be necessary to resolve this open question as ongoing hybridization between pariah dogs, village dogs and domestic dogs occurs in extant populations. Unfortunately, obtaining quality data from such ancient tropical specimens is likely to be challenging. In the next section, we consider organismal traits that may be hypothesized to change under the processes of taming and domestication. Where possible, we note how historical differences between taming and domestication may be seen in extant populations.

### Organismal level traits

Among domesticated mammals, dogs are considered the species that exhibit the full suite of features associated with domestication (Fig. 1). Most domesticated mammals, including dogs, tend to have smaller bodies than their wild counterparts, with smaller skulls that have shorter, wider snouts and shorter, lower jaws that make adult dogs look more puppylike than grown wolves do. Plausibly the observed reduction in body size under domestication reflects a shift along the continuum from selection for individual viability toward local selection for higher reproductive rate. Therefore, the shift in body size may have occurred as a response to the changed environmental conditions created around and within human habitations rather than the result of intentional selection by people. Other traits common among domesticated mammals, such as presence of depigmented fur and skin, a curly tail and floppy ears, are seen in dog breeds but are absent in the dingo. Here, we consider the organismal level traits relevant to the dingo as an untamed/feralized dog including skull morphometrics, brain size and seasonal breeding.

#### Skull morphometrics

Skull morphometrics have been used widely to distinguish dingoes from domestic dogs and hybrids [66, 73–75]. The morphometric method uses eight skull measurements in a canonical equation to establish a composite skull score. The status of a canine is established based on the composite score and the 95% confidence limits of each state. More recently, a broader set of 12 measurements was used to distinguish between a sample of posited dingoes known to per-date 1900 and similar-sized domesticated dogs [19]. Classification methods based on linear skull measurements have met with varying degrees of success, due in part to uncertainty over sample composition (i.e. purity of specimens) and the magnitude and patterning of variation in dingoes (i.e. Desert v Alpine). Dingoes generally show a broader and shorter skull, with a wider palate and shorter rostrum than do domesticated dogs [19, 73, 74]. The domestic dog features have been interpreted to be the result of paeodomorphism (retention of juvenile features in adults) associated with dog domestication [76–78]. Under a paedomorphic hypothesis, domesticated dogs (descendants) are considered to resemble wolves (ancestors) at a younger stage of development. The results of geometric morphometric studies, focused on the explicit 3-dimensional (3D) analysis of skull shape using landmark data, have challenged the idea that dogs are paedomorphic wolves. The short, broad skulls of domesticated dogs were concluded to be neomorphic, that is reflecting novel features which are not simply juvenilized variants of wolf morphology [79–81].

Recent work indicates that reduction in absolute and relative cranial length may be an early indicator of tameness [82]. Geiger et al. [82] collected longitudinal data for a population of house mice that experienced frequent exposure to humans without deliberate artificial selection, mimicking the early stages of tameness associated with the commensal pathway. Besides a reduction in head length, the population also displayed white spots of coat colour, a common feature among domesticated mammals. Therefore, tameness may result in a limited set of quantifiable traits that are distinct from the full suite of features associated with entering into a reciprocal pairwise relationship with humans, i.e. domestication [83].

Cranial landmark data have been used to tackle the question of how shape variation in the skull of wolves, dingoes and domesticated dogs is organized [84]. These data have specifically investigated the role of covariance between subsets of traits (modularity, [85]) in shaping cranial variation that is associated with domestication. The concept of modularity has received significant attention in relation to its hypothesized role in morphological evolution ([86, 87], and references therein). It reflects the idea that subsets of traits, modules, sharing strong connections with one another in a structure can evolve independently from other traits to which they are weakly connected, promoting the generation of morphological diversity. Based on 3D cranial landmark data, dingoes, domesticated dogs and their hybrids were found to share the same pattern of cranial modularity, and hybridization was not found to alter these patterns [88]. Of note, however, hybrids were found to resemble the cranial shape of dingoes most closely, which was distinct from cranial shape in wolves. Most recently, dingoes have been shown to be distinct from other canids in terms of cranial trait covariance patterns in the skull, representing an extreme version of the patterns recovered in the family [89]. This result has led to the suggestion that the domestication process in dogs may have taken advantage of flexibility present in the trait interaction patterns of ancestral forms, rather than re-patterning these associations anew [89].

Comparison of cranial growth trajectories in wolves and domesticated dogs with those from a sample of dingoes and pointing dogs has revealed that dingoes show a more similar growth pattern to wolves than to modern kennel breeds [81]. More generally, postnatal cranial growth differences between domesticated dogs and wolves appear at the earliest stages of postnatal ontogeny sampled, leading to the suggestion that differences in patterns between the two are likely to have arisen prenatally [81, 90, 91]. One potential area for future research is the examination of cranial growth patterns between Alpine and Desert dingoes, domestic dogs and hybrids. Such sampling of canids of known-age would permit assessment of differences in maturation and attainment of size/shape traits with age. Accelerated sexual maturation has been suggested to be a by-product of selection associated with high-output breeding regimes in domesticates [92, 93] or the result of provision of highly nutritious diet [94, 95]. Very little is known about maturation of brain tissue and craniofacial traits for dingoes, particularly at early stages of development when organogenesis is still ongoing and plastic responses to environmental influences, such as socialization, may result in measurable shifts in traits [81, 96].

Testing for selection in specific genes linked with known cranial functions is likely to be a fruitful area of research that will likely give insight into the evolutionary history of the dingo and its relationship with both wolves and domestic dogs. In a timely review, Schoenebeck and Ostrander [97] discussed the origins of dog skull shapes and highlight recent advances in understanding the genetics of skull morphometrics that can be extended to the dingo. For example, genome wide association studies have identified variation in the gene *bone morphogenetic protein 3* (*BMP3*) to be strongly associated with variation in skull morphology of domesticated dogs [98]. Wiener and colleagues [99] compared show- and hunting-type Labrador Retrievers from UK and found differentiation of genomic regions that included several genes associated with craniofacial development. Show-type Labrador Retrievers have slightly shorter muzzles and wider heads than do the hunting-type. The evolutionary allometry of rostrum length, has also been linked to the glutamine-alanine tandem-repeat ratio in *runt-related transcription factor 2* (*Runx-2*) in carnivores but it is not conserved among mammals in general [100–102]. Specific tests of selection may involve calculating differentiation metrics such as Fst or Population Branch Statistic (PBS) to test for significantly faster evolution in the cranial genes in the dingo [103, 104]. Subsequently the HKA test may be used to evaluate if these changes can be attributed to adaptive evolution [105]. These single marker tests will be complemented using haplotype-based tests such as EHH, iHS and XPEHH that are designed to identify positively selected loci [106–109]. Next, we consider brain size.

#### Brain size

Reduced brain size in domesticated as compared to their wild-living relatives has been observed for canids [110], fowl [111, 112], rodents [113], among others (see [90], for review). Further, feralized mammals have been shown to retain comparatively smaller brain sizes than their wild relatives [114, 115]. Plausibly, this reflects the functional outcome of selection on behavioural traits with regions associated with higher processing functions most markedly affected by size decrease ([116], and references therein). Brain size is heritable and has been positively correlated with survival and negatively correlated with fecundity [117–120]. Further, brain size predicts problem-solving ability in mammalian carnivores [121]. In a rare study of 45 wolves, 22 domestic dogs and 82 wolf × poodle hybrids Weidemann [120] examined the brain-body mass relationship and found wolf brain masses to be 29.8% greater than those of poodle brains. In F1 wolf × poodle hybrids, brain mass was intermediate to the two parentals, weighing approximately 16.3% less than that of wolves. Among F2 wolf × poodle hybrids brain mass showed segregation with approximately 30% of animals having brain weights like that of the parental wolves or poodles.

Due to the difficulties of directly measuring brain size, endocranial volume is frequently used as a proxy [122–124]. In a study of red deer on the Isle of Rum, Scotland, Logan et al. [119] used endocranial volume as a proxy for brain size and found positive correlations with lifespan and lifetime reproductive success. Isler et al. [123] compared endocranial volume from 3813 primates, at least 89% of which were wild caught, and found it did not differ between wild and captive/tamed animals, whereas body mass varied with living conditions. In contrast, the magnitude of variation in endocranial volume has been shown to be less for wild as compared to domesticated mink populations and was interpreted to reflect the lack of direct selective pressure on the brain in domestication events [125].

**Fig. 3:**
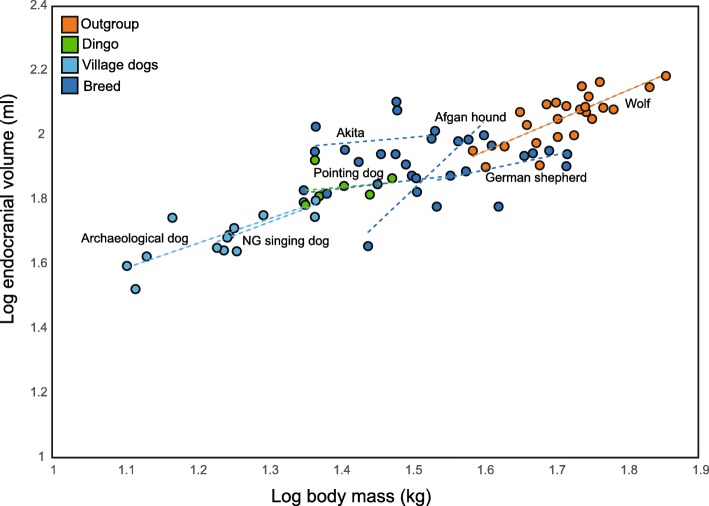
Double log plot of estimates of adult endocranial volume and body mass. Estimates were calculated from raw cranial landmark data provided in Geiger et al. [81]. Following Geiger et al. [81], breed refers to modern breed as recognized by kennel club standards, and village dogs refers to ‘premodern’ domestic dogs (NG = New Guinea). The latter are defined as populations that are geographically or culturally isolated from modern domestic breeds and that are situated in well-supported, basal positions on molecular phylogenetic trees [81]

To evaluate the prediction that dingoes may show brain sizes within the range of wild canids, we used published cranial landmark data [81] to extract external braincase measurements. The sample comprised adult representatives of wolves, ‘modern’, ‘premodern’ and ‘archaeological’ dogs [81]. Following Geiger et al. [81], ‘modern’ dogs, defined as breeds recognized by kennel clubs, were represented by the German Shepherd; ‘Premodern’ dogs were defined as populations that are geographically and/or culturally isolated from modern breeds and were represented by the Afgan hound, Akita, New Guinea singing dog, and dingo; ‘Archaeological’ dogs were Iron Age and Neolithic dogs from Switzerland (see [81]). Here, we calculated body mass estimates and endocranial volume estimates (as a proxy for brain size) using Carnivora-specific regression formulae [126, 127] for dingoes in comparison to a sample comprising wolves, breeds that are relatively similar to wolf skull morphology and pre-modern and archaeological domestic morphotypes (Fig. 3; Additional file 1). Considerable variation in endocranial volume is evident among canids, particularly among the modern breeds (Fig. 3). The dingoes in the sample fall largely along the same regression line as the village dogs, pointing dogs and wolves (Fig. 3), rather than showing a parallel shift along the y-axis, which would be indicative of smaller relative endocranial volume (as a proxy for brain size). In contrast, the Afghan hound and Japanese Akita show some deviation from the common allometric relationship, and the German Shepherds show relatively smaller brain sizes for similar body mass when compared to wolves. We conclude that the dingo appears to show similar brain size to modern breeds of a similar body mass, however, we do not know whether these dingoes were genetically pure or whether this may bias our analyses.

More detailed examination of brain morphology in Alpine and Desert dingoes is warranted. Notably, the extraction of virtual endocasts from computed tomography (CT) scan data (e.g. [128, 129]) would allow for the relative volumes of brain regions to be evaluated. Examining regions of the brain relating to sensory perception, that have been shown to differ in wild/domestic comparisons, would offer a framework for assessing how the dingo brain compares to that of modern domesticated breeds and the wolf. To explore the possibility of distinguishing between tameness and domestication, quantification of size differences in regions of the forebrain associated with the central nervous system role in tameness, the amygdala and other components of the limbic system [36], may be a promising start point. Next, we consider differences in seasonal breeding between wild canines and domestic dogs.

#### Seasonal breeding

The dingo and other wild canines differ from most domestic dogs in having a discrete breeding season and produce fewer pups per litter than do domesticated dogs [130–132]. Typically they produce one litter of 4 to 5 pups per year [130]. With the exception of the Basenji [133] and street dogs in Jaipur, India [134], domesticated dogs are continuous breeders and produce litters of 4 to 7 pups [132, 133, 135–137]. Seasonal breeding occurs in most wild mammals and is timed by photoperiod to coincide with seasonal abundance of food [130]. Wild dogs also reach reproductive maturity later than do domesticated dogs. It has been proposed that the absence of seasonal breeding in domestic dogs may be an adaptation to a niche created by permanent human settlements and their associated waste ([138] but see [134]).

One prediction of seasonal breeding is that reproductive organs will exhibit seasonal changes in traits such as size and function. Catling et al. [130] tested this prediction and observed significant seasonal changes in both male and female reproductive traits for wild and captive dingoes but not for domestic dogs. Male dingoes exhibit a significant, seasonal increase in testis size, prostate weight, semen volume and changes in testis histology that begins in January to March and peaks in April to May (Autumn/ early Winter in the southern Hemisphere) [130]. Female dingoes similarly display tumescence between April and July. Uterine weight increases significantly in April and peaks in May to June, coincident with females carrying foetuses. Female lactation increases in June and peaks July to August. In contrast, a significant seasonal pattern was not observed in male or female dingo × dog hybrids. Male hybrids showed no significant changes in male reproductive traits throughout the year and lactation was observed for a female hybrid in November [130]. Supporting the hypothesis that seasonality functions to restrict breeding to times when food is abundant, Catling et al. [130] observed that reproductive timing was delayed by 2-months in central Australian dingoes during a drought period.

The lack of a seasonal breeding cycle in domestic female dogs makes the timing of oestrus unpredictable. This unpredictability may cause males to maintain a continuous reproductive state. Domestic dogs, including free ranging wild dogs, have an opportunistic, promiscuous mating system in which male success may be decided by sperm competition. It is predicted that sperm competition will lead to selection for either greater sperm volume or more sperm and will affect testes size or sperm morphology. Woodall et al. [136] examined the reproductive structures of domestic dogs and dingoes and found a greater total length of the cauda epididymis in domestic dogs. The cauda epididymis functions in the maturation and storage of sperm [139]. Larger sperm storage volume may be an adaptation of male domestic dogs to unpredictable female oestrus. As dingoes have a shorter cauda epididymis it suggests that either it was never elongated, as in domestic dogs, or the increased length has been lost during feralization. Plausibly, the length of the cauda epididymis could be measured in well preserved archaeological dingoes to test whether it was never elongated, as in domestic dogs, or the increased length has been lost during feralization.

It is generally understood that photoperiod is the main factor that synchronises oestrus in many species, however, seasonality in oestrus has also been attributed to other regulatory factors, such as ambient temperature [140]. Despite this the regulatory mechanism of the oestrous cycle and male fertility at the cellular and molecular levels and the expression and function of genes in reproductive tissues are not fully understood. Future studies investigating the mechanisms underpinning the oestrus cycle and male fertility in dingoes and domestic dogs may be expected to give insight into the evolutionary history of these canids. Next, we consider sociability because it is hypothesised that communication through eye-gaze with humans was acquired by dogs during the process of domestication [141, 142].

#### Sociability

Domesticated dogs are skilled at sending and receiving communicative signals to and from humans. When encountering an unsolvable task in the presence of a human, domesticated dogs will exchange long, direct eye contact with the human while a wild wolf will not [143]. Dogs are also more skilled than wolves at interpreting human gestures [144]. Nagasawa et al. [145] studied gazing behaviour between wolves or dogs and their owners and found that wolves will make eye-contact more often but do not hold a direct eye-gaze while dogs hold a small number of long eye-gazes with their owners. Boitani and Ciucci [146] studied the social ecology of feral dogs in Italy. They found evidence to support the hypothesis that behavioural traits acquired during domestication, particularly lower levels of observational capacity and responsiveness associated with living in a ‘safer’ (i.e. human) environment, persist in feralized populations.

Reasoning that dingoes share an early domestication history with dogs, Johnston et al. [142] examined eye contact between dingoes and their owners. In contrast to the wolves tested previously, they found that dingoes initiate eye contact with humans but hold it for shorter times than were reported for dogs by Nagasawa et al. [145]. Johnston et al. [142] concluded that the motivation to make eye contact with humans likely evolved “*early in the domestication process*”, but the motivation to maintain prolonged eye contact with a familiar human may have evolved later. We suggest that this result is consistent with dingoes being tamed but not domesticated.

Domesticated dogs display a behavioural phenotype that includes playfulness, sociability, trainability, curiosity and attachment to humans. A screen for signal of positive selection in the domestic dog genome identified a 5-M base region on chromosome 6 that, in humans, is associated with Williams-Beuren syndrome (WBS). In humans WBS is a multisystem congenital disorder that is characterized by hypersocial behaviour. Structural variants of two genes, *GTF2I* and *GTF2IRD1* show a signature of positive selection in domestic dogs [147]. vonHoldt et al. [141] analysed this region further and observed that structural variants in *GTF2I* and *GTF2IRD1*, genes previously implicated in the behavioural phenotype of patients with WBS and contained within the WBS locus, contribute to extreme sociability in dogs. Future studies may examine sociability and *GTF2I* and *GTF2IRD1* variations in the dingo and domestic dogs. The specific test of the sociability assay is that dingoes should show the ancestral alleles and regulation of *GTF2I* and *GTF2IRD1* if the dingo is tamed and not domesticated. Reconstructing a gene to return to its ancestral function is considered unlikely.

In this section, we reviewed the organismal traits of skull morphometrics, brain and size, seasonal breeding, and sociability. We suggest that inclusion of dingo-dog hybrids and pooling of Alpine and Desert dingoes has caused considerable confusion with an unknown bias. We advocate that future morphological, behavioural and genetic studies should focus on including genetically pure Alpine and Desert dingoes. In the next section, we consider molecular and cellular traits focusing on the dingo. Currently phylogenetic analyses within Canidae use the inbred boxer genome (Canfam3) as a reference [28]. This a high-quality reference genome created from total of 31.5 million Sanger sequence reads, providing ∼7.5-fold sequence redundancy.

### Molecular and cellular traits

Alan Wilton, the father of dingo genetics, amassed a rich legacy of genetic information on this canid during his lifetime [46, 55–57, 68, 147–152]. Today, dingo genetic purity is still assessed using his methodology that is based on the frequency of microsatellite markers. The test now compares alleles of 24 markers in the canine subject against the frequency of marker alleles in populations of captive and wild dingoes that have allele frequencies different from that of domestic dogs. The allelic genotype of the tested canine is compared to that of a simulated dog-dingo hybrid. The comparison establishes the probability that the tested animal is a pure dingo rather than a canine that is 75% dingo and is scaled to the number of marker loci detected in the test; named the ‘3Q’ score. The final scoring of dingo purity takes into account the presence or absence of alleles found only in domestic dogs [148]. Despite his breakthrough genetic research Wilton’s work is essentially corroborative because all canids were sampled after Europeans arrived in Australia. Future studies aiming to extend the purity testing methodology should aim to include ancient samples known not to have hybridized with European dogs.

Here, we first review evidence considering the phylogenetic position of dingoes inferred using DNA from the mitochondrial genome [57, 68], Y-chromosome [55, 59, 72], genome-wide SNPs [147, 153] and short-read whole genome sequencing of an Alpine Dingo. [56]. The sequenced Alpine Dingo, named Typia, was bred in a colony that has been maintained at the Bargo Dingo Sanctuary in New South Wales, Australia for four generations. Consequently, he may be more inbred than wild dingoes. Fortunately, the Bargo sanctuary has focused on dingoes found in SE Australia he is likely a pure Alpine. We then consider metabolic genes that appear to be under positive selection and discuss the potential for the microbiome to compensate for organismal deficiencies in the host. The influence of the microbiome on the hosts’ survival and reproductive success is increasingly recognised [154].

#### Dingo molecular phylogeny

Molecular data do not clearly establish the phylogenetic position of dingoes. However, genetic as well as cellular and molecular traits are becoming increasingly available for canids and high-resolution comparative analyses between wolves, dingoes and domestic dogs can be expected within the next few years. Currently, the only consensus is that there are at least two dingo ecotypes and these are closely related to New Guinea singing dogs [46, 53].

First, we will consider inferences gathered from mtDNA. Savolainen et al. [57] sampled 582 bp of the mtDNA control region from 211 dingoes. These dingoes were selected based on similarity of appearance to dingoes, but were not tested to be genetically pure. Given the difficulty of identifying pure dingoes from dingo-dog hybrids [88] this sample is assuredly a mixture of mitotypes from pure dingoes and hybrids with an unknown bias. Still, there were 20 mtDNA types differing by at most two substitutions. Savolainen et al. [57] posited that dingoes have an origin from domestic dogs from south east Asia and were introduced from a single population of dogs “*possibly at a single occasion*”. Oskarsson et al. [149] used the same 582 bp of the mtDNA control region and concluded that the region could not definitively determine whether the dingo was actually a Neolithic item or a pre-Neolithic “*domesticate*”. More recently, Cairns [155] analysed 16,428 bp of mtDNA from 25 individuals sampled from five separate populations and a New Guinea singing dog. Each of the dingoes tested was characterised as having a maximum of one dog-like allele. Cairns [155] found 72 segregating sites and 21 haplotypes in the coding and RNA regions compared with just 6 segregating sites and 7 haplotypes in the control region. Combined these data demonstrate that the control region does not fully represent the mtDNA variation in the dingo and therefore is not expected to accurately reflect the maternal population history of the canid. In support of this hypothesis, Cairns and Wilton [46] showed that there were two distinct dingo mtDNA lineages that were not detected by an analysis of the control region.

Analyses of Y-chromosome data support the hypothesis that there are distinct lineages of dingoes [55, 59, 72] that may have arrived in Australia directly from Taiwan, independently of later dispersal of dogs through Thailand to Southeast Asia [59]. Ardalan et al. [55] sequenced 14,437 bp of the Y-chromosome from two captive dingoes and one New Guinea singing dog and then produced a haplotype network from “*non-homologous regions of the Y-chromosome*”. As homology is essential to systematics we find the resulting network difficult to interpret [156]. Sacks et al. [59] and Cairns et al. [72] genotyped 29 SNPs from pure dingoes and corroborated the presence of at least two dingo lineages in Australia that are geographically consistent with the morphologically characterised Desert and Alpine dingo populations.

Analyses of whole genome nuclear data are presently confusing and no clear consensus can be reached possibly due to difficulties in assigning homology, issues to do with long branch attraction, inclusion of an inbred captive dingo and the disparate algorithms and methods employed. An extensive genome-wide SNP survey of canids placed Typia the Alpine dingo in a basal domestic dog clade closely related to the African Basenji [147]. Somewhat unexpectedly, this tree suggests that other sight hounds including the Afghan hound, Egyptian Saluki, Greyhound and Whippet are derived. Wang et al. [153] mapped sequence reads from 58 canids to the reference and built a UPGMA tree based on the SNP’s (Fig. S4 in [153]). They report that the Alpine dingo and New Guinea singing dog cluster with the northern Chinese Chow Chow and these form a monophyletic group with the Cantonese Shar Pei and the Japanese Akita. In contrast, the African Basenji clusters with the FAMINGR, the Afghan hound and the Egyptian Saluki. In this tree the English Greyhound and Whippet sighthounds are derived. Subsequently, Wang et al. [157] conducted a principal component analysis for 1203 canids and highlighted a cluster of dogs that they suggest are closest to grey wolves (Fig. 2d in [157]). This group includes the African Basenji, Chinese Chow Chow, Cantonese Shar Pei, Japanese Akita, dingo and New Guinea singing dog, but not the Afghan Hound or the Egyptian Saluki. Notably, the principal component plot shows the dingo and New Guinea singing dog form a discrete cluster that is closest to Grey wolves. Fan et al. [158] included a multitude of wolves and constructed a maximum likelihood tree from whole genome SNP data. They did not include the Boxer, but found the African Basenji was the basal dog breed and the Alpine dingo is a derived domestic dog most closely related to two of the three Chinese indigenous dogs included in their study. The latter data suggest that dingoes were historically domesticated and are now feralized.

Freedman et al. [56] generated short-read genome-wide data from six canids. The preferred population tree suggested the Alpine dingo is basal to a dog clade that included the African Basenji and the Boxer reference. This population tree can be interpreted as the tamed dingo ancestors were *unconsciously* selected by humans after the split from wolves and then domestic dogs *artificially* selected, possibly in multiple places [41]. An alternative explanation is that canid domestication occurred immediately after divergence from the wolf.

**Fig. 4:**
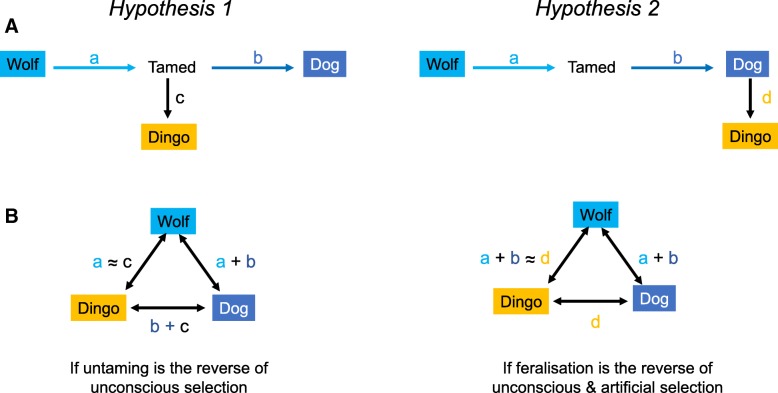
Simple predictions of the sets of genes that may be expected to be under selection if dingoes are now untamed (Hypothesis 1) or if they are feralized (Hypothesis 2). A. Illustrates the sets of selected genes on each lineage including unconscious selection (a), artificial selection (b), untaming (c) and feralisation (d). B. Illustrates the sets of genes that may be seen when conducting pairwise tests of selection. Note here, we do not know whether the full set of genes involved in taming is required for untaming (a ≈ c). Further, we do not know whether feralisation involves the full set of genes involved in unconscious plus artificial selection (a + b ≈ c)

Future studies should aim to complete long-read assemblies of a wild (non-captive) dingo and compare this genome to a similarly constructed genome from a domestic dog. This will enable genes under selection (Fig. 4), structural variants as well as SNPs to be included in future analyses. Setting a new benchmark, Kronenberg et al. [159] coupled long-read sequence assembly and full-length complementary DNA sequencing with a multiplatform scaffolding approach to characterized lineage-specific and shared great ape genetic variation ranging from single- to mega-base pair-sized variants.

#### Metabolic genes that appear to be under positive selection

It is hypothesized that duplication of *Amy2B* gave domestic dogs an evolutionary advantage in adapting to a novel, human-provided, starch-rich diet. Amylase is the digestive enzyme needed to digest carbohydrates. In support of this, it is that estimated serum amylase enzymatic activity increases by 5.4% with each additional copy [160, 161]. Ollivier et al. [162] investigated the timing and expansion of the *Amy2B* gene in Europe and Southwest Asia and found ancient dogs had between 2 and 20 diploid copies of the gene. They suggested that selection for the increased *Amy2B* copy number started more than 7000 years BP. There are 2 to 34 copies of *Amy2B* in modern dog breeds while the genomes of wild wolves contain 2 to 8 with about 60% of wild samples having only 2 copies.

**Fig. 5:**
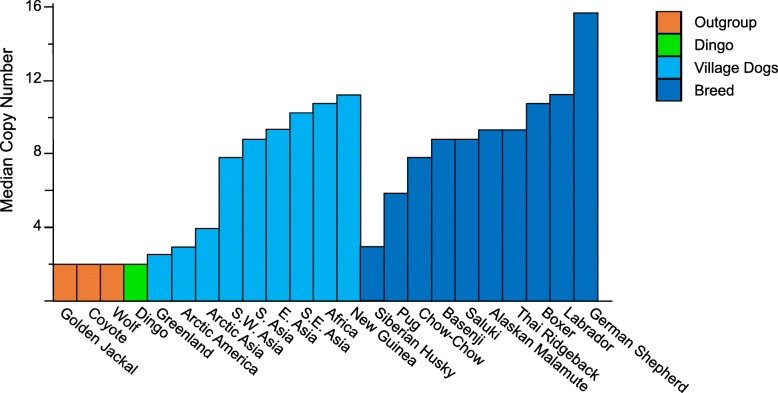
Copy number variation at amylase (*AMY2B*) locus. Median copy number variation (CNV) at *AMY2B* obtained and plotted from [163]. Note that not all the breed dogs listed in [163] are included. Rather we have focused on those related to this article and included some well-known breeds

Freedman et al. [56] found no expansion of the *AMY2B* locus in the Alpine dingo they sequenced. Arendt and colleagues [163] corroborated this result (Fig. 5) and found 37 dogs carried the ancestral *AMY2B* copy number of two. Of these 22 were dingoes, 10 were indigenous sled dogs and two were dog breeds of Chinese origin (one of four Chow Chows and one of three Pugs). There are at least three possible explanations for the low *Amy2B* copy number in dingoes (Box 4). We do not include the possibility that dingoes hybridized with wolves in Australia as wolves have not been found in Australia. However, modern day hybridization has probably influenced *AMY2B* copy numbers in other canids. New Guinea singing dogs, the acknowledged sister group to the Australian dingoes, have 9–22 copies of *AMY2B* [163]. Given the chequered recent history of these dogs in Europe and North America, and the genetic sampling of dogs from the captive population in North America, hybridization with domestic dogs is possible. Assaying historical samples or recent specimens collected from New Guinea would help resolve this conundrum. Hybridization of Arctic dogs, particularly sled dogs, with wolves is also possible. In the United States, over 100,000 wolf-dogs exist [164].

Future studies may consider assaying the influence of *AMY2B* on food preferences and starch digestion in dingoes and domestic dogs. Rao et al. [165] tested similarly raised and kept wolves and dogs in two different food choice tasks, a classic two-choice task and a multiple-choice paradigm. They found that wolves and domestic dogs did not differ in their preference for meat over kibble in either paradigm. However, wolves (but not dogs) choice patterns were affected by satiation, with wolves being less “selective” when hungry. An alternate study design may be a food preference test. Hewson-Hughes [166] performed diet selection studies in five domestic dog breeds (papillon, miniature schnauzer, cocker spaniel, Labrador retriever, and St Bernard) to determine whether they regulate macronutrient intake. Using nutritional geometry, they show that the macronutrient content of the diet was regulated to a protein: fat: carbohydrate ratio of approximately 30%:63%:7% by energy. Such behavioural macronutrient preference studies should be able to distinguish recent untaming and feralization, but may not distinguish historical untaming from ancient domestication and feralization. Importantly, studies testing whether dingoes and domestic dogs have the same macronutrient preferences have potential to give insight into foraging of these canids in Australia.

Several additional regions of the dog genome appear to be targeted by selection during domestication and include genes that are associated with digestion and energy metabolism [161]. Currently, it is not known whether these same genes are under selection in the dingo, but the reversal of genetic pathways during feralisation is expected to be rare. As such, the historical signature of domestication in the genome is expected to remain. As an example, two members of the ATP-binding cassette transporters superfamily, ABCG5 and ABCG8, which have pivotal roles in the selective transport of dietary cholesterol appear to be under selection in domestic dogs [157]. Plausibly these changes are linked with drastic alterations in the proportions of plant food, relative to animal food, consumed by canids during the domestication process [167]. The diet of wolves, is predominately composed of animal protein with very little intake of vegetal matter [167]. Dingoes have been characterized as possessing a flexible and generalist diet that varies with bioclimatic zone, reflecting variation in abundance and composition of reptile and mammal prey [168]. In contrast, domestic dogs eat human food, starchy foods, protein and fat. Dietary changes may also influence the gut microbiome, which is an important if poorly understood feature that has been shown to influence organismal and cellular traits [169–171].

#### Gut microbiome

Taming and domestication likely involved dietary shifts, which may influence the microbiome. The extent to which species-specific faunal communities may override attempts at assessing domestication hypotheses is not clear, likewise it is not clear whether a wolf-like microbiome could be re-established by untaming or by feralisation. However, linking the microbiome with amylase copy number study (Box 4) in a dietary manipulation study may enable a clear interpretation of the result.

Diet shifts involve changes in composition (e.g. shift from exclusive animal protein to mixed diet), variety (e.g. singular dietary source compared to diverse food intake) and level of contact with the environment (e.g. soil). These factors have been implicated in the maintenance of microbial diversity in tamed animals but decrease in biodiversity observed for comparisons of wild and captive mammals [172]. Wild-caught rodents have been shown to retain the majority of their native gut flora after being held in captivity and fed commercial food for substantial time periods [173]. Metcalf and colleagues [174] found that microbial diversity was greater in the gut of wild Przewalski’s horses (*Equus ferus przewalskii*) than in herded domestic horses (*E. f. caballus*) that inhabit adjacent natural grasslands. Evidence from additional studies further supports taxon-specific responses to changes in gut flora. In a comprehensive survey of 41 mammalian species from six orders McKenzie and colleagues [175] observed significantly decreased gut microbiota diversity and composition in carnivores and primates. The fall in diversity was not universal across sampled mammalian groups, but herbivory was generally associated with a more stable gut flora and a protective role of fibre in the diet was proposed [175].

During domestication, humans have changed the environment that an animal interacts with, often by reducing its complexity, increasing cleanliness, alerting stress levels and changing conspecific interactions. Wu et al. [176] found 15 bacterial species that differed in abundance between captive wolves and dogs. Furthermore, a metagenomic analysis of wolf and dog gut microbiotas revealed differences in microbial species and genes related to starch and cellulose digestion [177]. Lyu et al. [177] suggested that the gut flora of dogs reflects adaptation to a diet of human food, most notably the presence of starch combined with a low intake of animal protein. A significant difference in the abundance of genes encoding glycosyltransferase family 34 (GT34), carbohydrate-binding module family 25 (CBM25), and glycoside hydrolase family 13 (GH13) between the gut microbiota metagenomes of wolves and domestic dogs and suggests there are important differences in carbohydrate metabolism between these taxa. Based on the low amylase copy number of wolves and dingoes (Fig. 4) as well as their dietary preferences [168] we predict that the microbiome of dingoes will be more similar to wolves than domestic dogs.

In humans the gut microbiome is influenced by lifestyle factors including diet and physical and emotional stress [178]. An important and open question is the extent to which dietary changes may induce transient shifts in the abundance and composition of gut flora communities and how these may translate into fitness costs, such as impaired digestive and immune function, incurred by the host in instances of drastic shifts in diet. Understanding how well the host may tolerate drastic dietary changes, and the extent to which an impoverished or unbalanced gut community may be retained in instances where that dietary shift is permanent, would be a valuable avenue for future study in cases where domestics have returned to the wild.

In this section we have discussed hypotheses concerning the phylogenetic position of dingoes, metabolic genes that appear to be under positive selection in the dingo and the potential for micronutrient compensation by the gut microbiome. Clearly, however, incite from other species can also direct and focus future research. Sequencing and assembly of the red fox genome enabled the comparison of tame and aggressive populations that were developed over five decades of selection for behaviour [179]. One positional positional candidate gene for tame behaviour was identified as *SorCS1*. This gene encodes a trafficking protein for AMPA glutamate receptors and neurexins and suggests a possible role for synaptic plasticity in fox taming. A wolf reference genome is also now available [180]. The authors concluded that studies aiming to study variation within wolves and their relationships to dogs should use the de novo assembled wolf reference genome. Here we suggest that future studies aiming to study variation within dingoes and their relationships to wolves and dogs should aim to use a de novo assembled dingo reference genome from a wild caught animal.

## Conclusions

Policy makers walk a tightrope between competing interests. In the case of the Australian dingo influential pastoralists and members of the mining community are declaring that the dingo is simply a feralized dog. And, as a feral dog it can be trapped or shot on sight. We assert that the evolutionary and domestication history of the dingo are far from settled and much research is still needed. We further propose that caution needs to be exercised in labelling the dingo as feral, without compelling data, because extinction of the canid is becoming increasingly likely both from political pressures and hybridization with domestic dogs [150].

Future studies of quantifiable traits associated with genetic variants will clarify the position of the dingo in canine evolution. Plausibly, the dingo occupies a unique position between wolves and modern domesticated dogs that can be leveraged to understand the domestication process. Alternatively, dingoes are feralized domestic dogs whose ancestors were domesticated in SE Asia. There is no evidence of continued long term selection of dingoes in Australia that has resulted in domestication, though there may have been episodic attempts at breeding by Aboriginal people [39, 63]. We have identified traits and genes that are hypothesized to differentiate wild from domesticated dogs and that can be explored in the emerging body of dingo genome sequence. We propose that future morphological, behavioural and genetic studies should focus on including Alpine and Desert dingoes that are demonstrated to be genetically pure and not dingo-dog hybrids. Recombination can lead to the introgression of specific genes into populations and these can affect the behaviours of animals in complex ways [181].

We suggest that incorporating ancient DNA data as well as understanding the mechanisms involved in amylase copy number expansions may be pivotal to understanding the evolutionary history of the dingo. This will most likely involve collaborations between scientists in different fields. Cementing the future of the dingo will necessarily also involve discussions between the Aboriginal people, policy makers and conservationists.

Box 1 In the view of Charles Darwin [23] there are two steps to the process of domestication *“Methodical selection is that which guides a man who systematically endeavours to modify a breed according to some predetermined standard. Unconscious selection is that which follows from men naturally preserving the most valued and destroying the less valued individuals, without any thought of altering the breed; and undoubtedly this process slowly works great changes. Unconscious selection graduates into methodical, and only extreme cases can be distinctly separated; for he who preserves a useful or perfect animal will generally breed from it with the hope of getting offspring of the same character; but as long as he has not a predetermined purpose to improve the breed, he may be said to be selecting unconsciously*”.

Box 2 Gonzalez [43] defines the dingo-type as “*medium body size, well proportioned rib cage, slightly long back and long legs the head appears pear shaped when looked from above and the neck is strong and of a medium length, the muzzle is triangular and relatively long eyelids are lightly slanted the tail is often curled up, very frequently carried over the hips, sometimes in an almost closed loop, although in some cases can appear hooked or pendant, and it is usually smooth or feathered, rarely bushy ears are of a medium size, erect, triangular and wide at the base. Coat colours are variable with ginger tones (red, yellow and sandy) dominating specimens displaying this colour phase often have two or more white feet, a white tail tip and sometimes white chest and throat areas, and more rarely a white muzzle sable specimens are also relatively common as well as piebalds, black and tans and blacks light grey and full white specimens are uncommon*”.

Box 3 Gonzalez [43] defines the village-type dog as “*rather similar to the dingo type but lighter, about three quarters of its size, and much more gracile, limbs are not as well muscled and the chest tends to be narrower and shallower ears are longer, the tail is usually smooth or feathered but never bushy and is carried almost without exception high over the rump, coat colour is as variable as in the dingo type*.”

Box 4 Possibilities for the low *Amy2B* copy number in dingoes 1. Dingoes never acquired the *AMY2B* duplication.

2. Dingoes lost the duplication and now have a single functional copy. A simple prediction from this hypothesis is that remnants of the *AMY2B* duplication are expected to be present in the dingo genome, unless they were precisely excised.

3. There have been multiple independent *AMY2B* expansions. This hypothesis predicts that expansions may have occurred independently in domestic dog breeds and therefore breeds that are paraphyletic relative to the dingo may not show the same expansion pattern.

## Additional file

Additional file 1: Endocranial volume and body mass values used to produce Fig. 3. Raw landmark data are available from [81]. (XLSX 111 kb)

## Abbreviations

*Amy2B*: Pancreatic *amylase*
BP: Before present
CNV: Median copy number variation
HPD: Highest posterior density and are a form of credible interval (the Bayesian analog).
mtDNA: mitochondrial DNA
SNP: Single nucleotide polymorphism
UPGMA: Unweighted Pair Group Method with Arithmetic mean
WBS: Williams-Beuren syndrome
